# A qualitative feasibility study of a prototype patient-centered video intervention to increase uptake of cancer genetic testing among Black Americans

**DOI:** 10.1186/s40814-024-01482-8

**Published:** 2024-04-11

**Authors:** Katherine Clegg Smith, Rachel Grob, Michelle McCullough, Betty May, Emily Warne, Amanda Matchette, Avonne E. Connor, Kala Visvanathan

**Affiliations:** 1 Sidney Kimmel Comprehensive Cancer Center, Johns Hopkins Bloomberg School of Public Health, Baltimore, USA; 2 Johns Hopkins Bloomberg School of Public Health, Baltimore, USA; 3 Department of Family Medicine and Community Health, University of Wisconsin Madison, Madison, USA; 4 Wellspan Health, York, USA

## Abstract

**Background:**

Health advances due to developments in genomic medicine are unequally experienced in the USA; racial differences in the uptake of genetic testing are one factor in this disparity. In collaboration with Black patients and diverse health care providers, we are developing a patient-centered video intervention to increase cancer genetic testing among eligible Black Americans. The objective of the pilot work is to explore the acceptability of and support for the intervention and key content components.

**Methods:**

In order to create a patient-centered video intervention prototype, we conducted a targeted, secondary analysis of 47 coded transcripts from video-taped qualitative interviews with people with a known genetic or inherited cancer risk. The review focused on decision-making, testing experiences, and perceived value of genetic testing. We subsequently generated a 15-min video montage of content from 9 diverse (age, gender, race) participants. We used the prototype video as prompt material for semi-structured interviews with 10 Black patients who had undergone genetic testing in the last 2 years and 10 racially diverse providers (genetic counselors, a nurse, and medical oncologists) who provide management recommendations for high-risk patients. Interviews sought to understand the acceptability of a video intervention to enhance informed decision-making by Black patients and key elements for intervention efficacy.

**Results:**

Study participants were generally positive about the prototype video and provided guidance for intervention development. Interviewed patients prioritized perceived authenticity and relatability of video participants. The presentation of patients’ perspectives on testing, their experiences of testing, and the benefits of having test results were all seen as useful. The benefits of testing for self and family were identified as important considerations. Privacy concerns and science skepticism were identified as germane issues, with guidance to present barriers to testing alongside possible solutions. The inclusion of clinicians was seen as potentially useful but with caution that clinicians are not universally trusted.

**Conclusions:**

Study findings provided critical input for the creation of a professionally produced, tailored intervention video for a randomized clinical trial with Black Americans to evaluate the influence on uptake of genetic testing. The interviews suggest the acceptability and potential utility of an authentic, realistic, and tailored, patient-centered video intervention to increase consideration and uptake of genetic testing.

## Key messages regarding feasibility

- Previous research has demonstrated the impact of video interventions on increasing knowledge and awareness among diverse populations, but the use of culturally tailored patient-based videos to improve the uptake of cancer genetic testing has not been extensively evaluated. It was not known whether a patient-based video about genetic testing would be acceptable to Black patients who are eligible for testing or health care providers who are involved in testing.
- This research uses available video content of actual patients talking about genetic testing experiences as prompt material for patient and stakeholder input for intervention development. Qualitative interviews conducted after showing patients and heath care providers the prototype intervention material highlighted authenticity, discussions of community norms, and experiential content as key feasibility components in the design of a future intervention. Diverse and reflective representation of Black patients who have undergone genetic testing was also highlighted as an important consideration in intervention development.
- This qualitative study demonstrated support for testing a patient-centered intervention video and provided important guidance for the development of a patient-based intervention video. Based on these findings, we have developed a professionally produced, patient-centered video intervention focused on the experiences of Black Americans to encourage the uptake of genetic testing. The pilot intervention also informed the decisions to include enhanced biographical context in the intervention video and ensure content that balances patients’ positive experiences with personal and community concerns and barriers to testing.

## Background

Rapid advances in genomic medicine are enabling a more personalized approach to the early detection, prevention, and treatment of individuals at high risk for cancer(s) or diagnosed with cancer. Having access to knowledge of personal cancer risk such as genetic testing can enable individuals to take part in shared decision-making with providers regarding cancer treatment, early detection, and risk reduction. There is also now evidence demonstrating a survival benefit for some of these preventive strategies among individuals with a hereditary predisposition [1]. Genetic testing can also help reduce cancer risk and promote early detection of cancer among family members. Despite an increase in our understanding of the potential value of genetic testing for individuals and families, uptake of testing continues to be significantly lower among Black patients with and without cancer [2–4]. Factors contributing to the disparity in uptake include unequal access to health care, lack of awareness, experiences of untrustworthy medical systems, fear of genetic testing, and unequal recommendations for genetic evaluation [5, 6]. In order to reduce disparities and create more equity related to prevention and treatment, there is an urgent need for innovative targeted interventions to help reduce barriers and improve uptake of testing among Black Americans through facilitating access, increasing awareness, reducing fear, and addressing concerns that lead to mistrust.

In 2018, the National Academies of Science held a workshop on improving disparities in access to genomic medicine [7]. A common theme throughout the workshop was that disadvantaged patients preferred to be educated in genomics from someone they know (i.e., their primary care provider, someone else in the family, another person who has the same condition) [7].

Provider-based educational videos about genetic testing are one response to such preferences and have been shown to increase patients’ knowledge and awareness of testing, decrease negative attitudes, facilitate information sharing, and increase intent to undergo genetic testing [8–10]. There is also evidence for increased uptake of same-day testing after exposure to such videos [10]. In non-cancer settings, there is some evidence that culturally tailored educational videos can impact participation, knowledge, trust in medical researchers, and willingness to participate in clinical trials among Black patients [11]. For instance, a video delivered by a diverse group of trained health educators was found to improve biospecimen donation compared to an educational brochure [12]. Despite studies demonstrating a positive impact of video interventions on increasing knowledge and awareness of individuals, the use of culturally tailored videos to improve the uptake of cancer genetic testing has not been extensively evaluated. Moreover, the literature is sparse in relation to studies that have assessed the use of patient narratives to effect change in the uptake of recommended health services. We see an opportunity to develop and test a targeted patient-centered video around experiences with genetic testing to evaluate whether this form of intervention can increase genetic testing uptake among eligible Black Americans.

## Methods

**Fig. 1:**
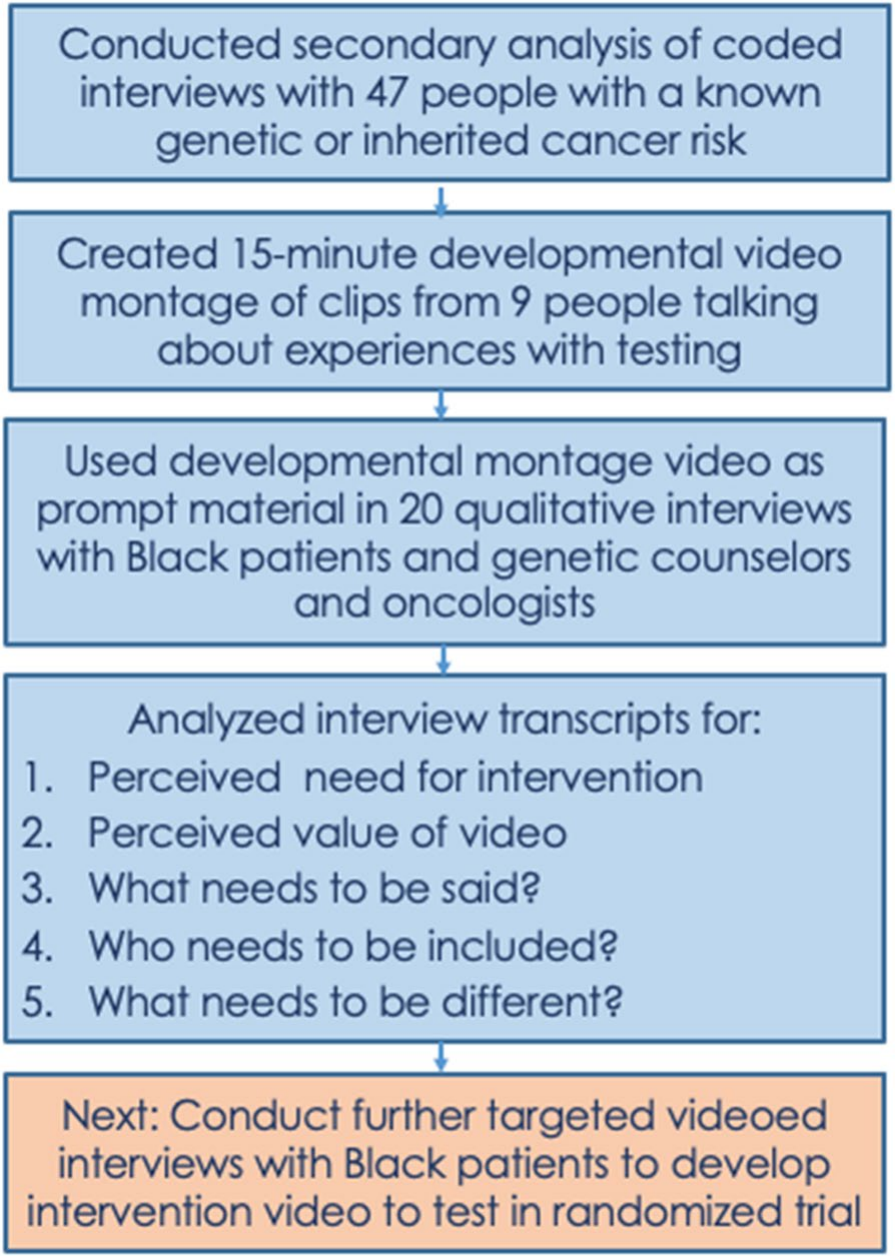
Creating and testing the prototype of a patient-centered video intervention

The development of the patient-centered video intervention is being undertaken by a diverse and interdisciplinary team of investigators and advisors that include experts in medical oncology, cancer genetics, genetic counseling, cancer epidemiology, disparities, cancer risk communication, qualitative methods, and patient health experiences (see Fig. 1). The first phase of the developmental research was the creation of a 15-min prototype video using data available from a recently completed, video-based qualitative interview study (https://www.healthexperiencesusa.org/Cancer-Risk-That-Runs-in-Families/overview).

**Fig. 2:**
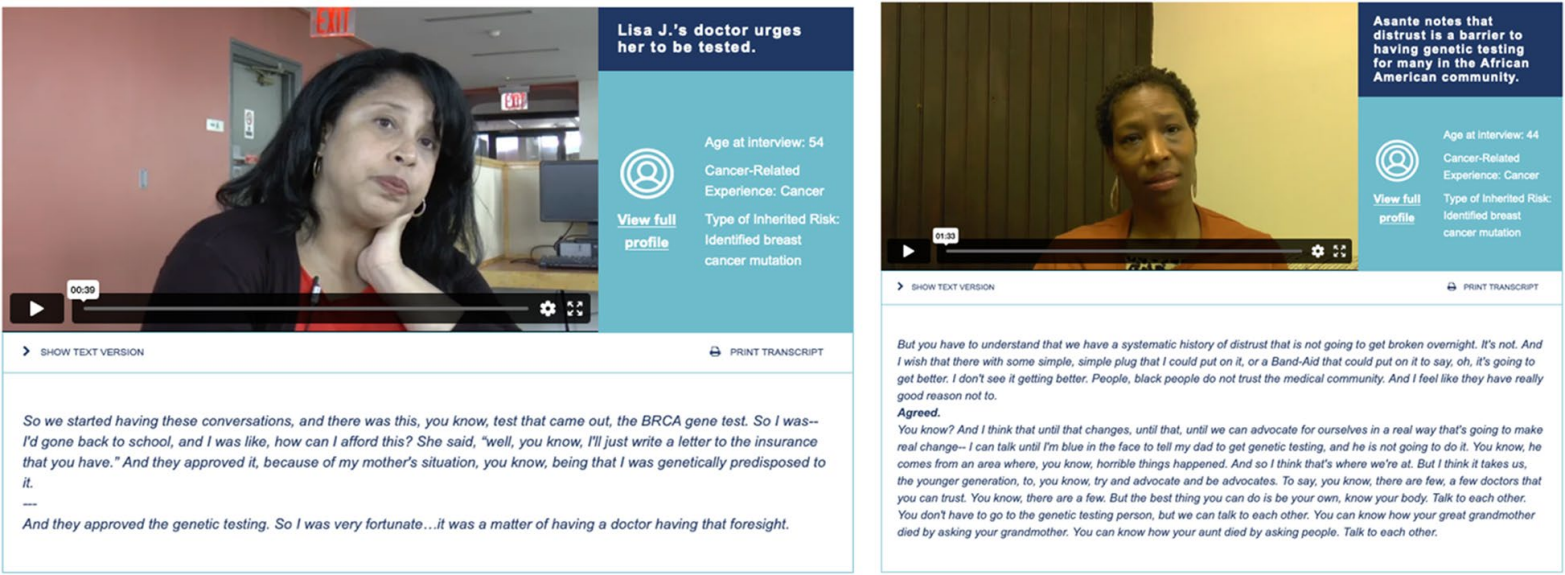
Examples of patient content in prototype video

The prototype video’s content was compiled from a review of transcripts from 47 qualitative interviews with people who had a known genetic or inherited cancer risk, some of whom also had their own cancer diagnosis. Consent for the prior study included an acknowledgment that video materials would be publicly available and provided an explicit agreement to future use of video segments on the Healthexperiencesusa.org website and for research, educational, and health system improvement. Several of the study participants in the prior qualitative video study were Black, but most were White. We reviewed all transcripts and prioritized the perspectives of the Black participants in relation to learning about their cancer risk due to a hereditary predisposition, their decision-making around genetic testing, and testing experiences. From this review of the existing interview data, we used a priori criteria considered key for intervention development and identified segments in which interviewees were talking about considering genetic testing, undergoing genetic testing, and communicating with their family about genetic testing and life following a decision to either test or not test. Each interview was read by at least 3 members of the research team the medical oncologist with expertise in cancer genetics, a qualitative researcher who had been involved in the initial study, and the study coordinator. The research team compiled all relevant pieces of the videoed interviews (from all participants) from which we created a 15-min prototype montage video. We assembled the video by selecting key segments to create video subsections, giving primacy to the representation of Black participants. The prototype video included 9 interviewees, of whom 4 are Black. In Fig. 2, we include 2 example screenshots of patients and content included in the prototype video.

The prototype video was used as prompt material in 20 qualitative stakeholder interviews: 10 interviews with Black patients (7 women and 3 men, aged 33 to 56) who had undergone genetic counseling and testing for cancer within the last 2 years and 10 with health care providers (3 medical oncologists who provide management recommendations for high-risk patients, 6 genetic counselors, and 1 oncology nurse). Patient interviewees were patients of one of the clinicians on the study team, and providers were identified through professional networks and snowball sampling. Recruitment occurred through personal outreach from members of the study team known to participants who explained that the goal is to develop a patient-centered intervention to increase the uptake of cancer genetic testing by Black Americans. The provider interviewees were a mix of Black (*n* = 5) and non-Black participants (*n* = 5). Given the small number of Black oncologists and genetic counselors currently working in the US health system, we felt it was important to show the montage to a diverse group of providers with a focused discussion on the needs of Black patients. The qualitative interviews were conducted by the first author who is a White woman and a highly experienced, PhD-trained qualitative researcher. The interview was centered on engagement with and reactions to the prototype montage video. In the consent process, all participants were made aware that our goal is to develop a video intervention to facilitate Black Americans’ informed decision-making related to genetic testing.

Given that this research was conducted in 2021–2022, with COVID precautions still prominent, all interviews were conducted via Zoom with interviewees located in their homes or workplaces depending on their preference. After a brief introduction, participants were shown approximately 10 min of video, and then the video was stopped, and they were asked for their overall reaction as well as targeted questions about the representation of factors related to decision-making about genetic testing, barriers to testing, and the testing process. Following this feedback, the second segment of the video (about 5 min) was then shown, and again, general reactions were sought followed by questions related to the content of the video, namely barriers and values to genetic testing, communicating about testing with family members, and practicalities of getting testing. We also asked for opinions about the content and structure of a future video intervention. How could what we showed them be improved? Participants were also asked about whether patient-centered videos were an appropriate intervention strategy, and if so, the preferred voices and messages to be included. We also asked for thoughts about what information could be important to improve the chances of uptake of genetic testing within the Black community.

Interviews lasted between 30 and 40 min and were transcribed with the assistance of the transcription feature of Zoom; each automated Zoom transcript was subsequently reviewed and edited by a member of the study team using the interview audio file. Given that our goal in this project is highly pragmatic—to develop a pilot video intervention—the analysis was largely descriptive and pragmatic, rather than theoretical. The first stage of the analysis involved creating a summary document for each interview organized around five key topics:

- Why do people test?
- What are the barriers to testing?
- What did people like about the montage video?
- What did people feel needs to change about the video?
- What is the potential value of a video-based communication intervention such as this one?

The final analytic step undertaken for the process of intervention development was for another member of the study team to re-review the segments of the interview transcripts related to each of the five topical areas using a constant comparative process, with a focus on comparing patient and provider responses, to identify areas of convergence as well as outlying perspectives. The initial analysis was then shared with the broader team for analytic input and refinement. No qualitative software was necessary for this analysis.

## Results

Overall, interviewees expressed strong support for an intervention to promote informed choice around genetic testing that is focused on patient-centered videos. Patients and provider interviewees both saw value in a patient-centered video conveying authentic experiences and reflections on genetic testing. Patient interviewees commented on the value of people sharing “real-life experiences,” and some said that it was particularly important that this should come from Black patients. They remarked that they found it reassuring to hear people talk about the testing process as simple and hearing that others saw it as important. They expressed that these would be compelling messages in an intervention for Black patients. One patient did, however, remark that this type of intervention would not have been valuable for him because he had such strong support from his medical team and community for testing, and did not therefore experience a need for it.

One of the video clips that mentioned “breaking the silence” within the Black community about cancer and inherited risk particularly resonated with interviewees (patients and providers). The video intervention was seen as a potentially effective way of encouraging or even modeling talking about cancer and medical history in a way that is not seen as common in the Black community. Below are illustrative examples of patients expressing support for the messages being shared in the video, with an emphasis on the inclusion of Black perspectives. Health care providers were also highly supportive, emphasizing value in the variety of perspectives and emotions being shared.

**Table:** 

**Interview extracts about the value of a patient-centered video intervention**	
“Adults absorb information and relate to people’s experiences, so it’s a beautiful thing that you have people sharing in that way.” (Patient 7)	
“I definitely think it’s important to keep the piece that the first woman talked about breaking the silence in the Black community.” (Patient 6)	
“I thought the content was really good. Each of the participants shared a different aspect or perspective on the genetic testing experience. People talking about how they were explained the pros and cons of testing, which I think is a big question for patients. And, how they felt about waiting for the test results…I thought that the video did a good job of showing the spectrum of thoughts around genetic testing and having a genetic mutation.” (Genetic Counselor, Provider 6)	

Patients and provider interviewees both saw the presentation of people’s reasons for testing as key to a patient-centered video intervention. Both noted that it is helpful for people considering testing to hear people talk about why they decided to test and what benefits it has had for them and that there is also value in presenting people’s rationales for not testing, or not testing immediately. Participants felt that a video intervention should support an informed decision around testing, rather than to push one towards a particular choice. The rationales of testing to facilitate preventive efforts for self, and also to serve one’s family and future family, were highly salient.

**Table:** 

**Interview extracts about why people test**	
“The idea that there are many cancers, given the evolution in cancer research that are very curable if they are detected early and having the ability to be screened more…” (Patient 5)	
“One thing about me, I worry about my kids and grandkids… its important to find this stuff out now, because we can help them.” (Patient 2)	
“I think that if it could be presented in a way that makes us feel like, you know, this is a good thing for us. This is the thing that will help us, help our community – our kids, stay on the earth longer, I think that is something that people would be more interested in. I think African Americans have more mistrust in medical professionals… and if you make it about them and their kids – in our community we are very big on making sure our kids are good.” (Patient 4)	
“You have to do the positive and negative, to make sure they get the whole picture…You have to inform – you can’t just give all the good, you have got to give the bad, too… Both sides are needed.” (Patient 2)	

In reflecting on the video, interviewees described several key barriers to testing as important topics to include. Some of these barriers were relatively generalized (e.g., financial constraints or fear of needles). Others were tied to historic injustices perpetrated on the Black community and the resulting mistrust of science, as well as the potential for ongoing discriminatory practices specifically tied to genetic testing.

**Table:** 

**Interview extracts about barriers to testing**	
“There is just a little conspiracy around science in general … Something that you would be able to address in this space … probably in terms of privacy and how the information would be used…its not shared in larger ways, if there is a timeframe day that you keep it.” (Patient 1)	
“I have a lot of patients who say that their children are young and is this going to come back to them? Are they not going to be able to have life insurance? Nobody in the video talked about that.” (Genetic Counselor, Provider 6)	
“One of the things that… got talked about by the very first person that spoke to the general sense of trust and how information is used… It was interesting for me to discover that insurance companies can take this information and make decisions about what you can and can’t have access to on the basis of testing, and that, I think, is deeply concerning.” (Patient 5)	

Relatability and representation were mentioned as key to the effectiveness of the video, although there was no agreement as to whether all videoed patients need to be Black in an intervention targeting a Black audience. Among patients, there was a feeling that it was helpful to see people “like them.” While most interviewees said that the video should be entirely Black patients (and some felt this strongly), others either felt that this was not critical or saw value in including non-Black voices. Several interviewees pointed specifically to the need for diversity of representation within the Black community. Diverse representation by age was identified as important by genetic counselors. Both younger people (who are positioned well for prevention) and older people (who may be key to understanding family risk) are key target groups for genetic testing from the point of view of providers and should be represented. Participants told us that it is important to have a good representation of men in the video, as men can be challenging to engage in relation to testing. One oncologist commented that it was helpful having people in the video who took a long time to decide to test (even years), as this may be empowering for patients who have known about testing for some time and may be concerned that they have delayed too long.

**Table:** 

**Perspectives on who should feature in a video intervention for Black Americans**	
[About including a patient who waited a long time to get tested in a video intervention] “I feel like it makes them feel like okay, well, even though I didn’t do it right away, its okay to go ahead and do that.” (Medical Oncologist, Provider 7)	
“One thing I can say is that it would help me if it was all African Americans.” (Patient 3)	
“I would say the majority of participants should be [Black], but I don’t think 100% because having different perspectives doesn’t hurt anybody…I think including other people makes it less of a stigmatized isolated event… we are all in the same boat. We are all struggling with the same issues.” (Patient 6)	
“Its always nice to hear directly from the patients about their experience. I do think I want to know more background information about each person. To make it even more personable… you always want to know who do I relate to? Whose situation is similar to mine?... Since the video is for African Americans, I was wondering why there were non-Black patients in the video.” (Genetic Counselor, Provider 4)	
“The people that you have in the video all look to be of different ages, gender mix, ethnicity mix – all of that matters.” (Patient 5)	

Patients described appreciating seeing other people like them discuss genetic testing as a mechanism to reduce risk by facilitating early detection. The age of video participants was specifically pointed to as a relevant factor; young people need to hear other young people’s rationale for testing. Patients also felt that describing possible benefits for children and future generations could serve as powerful messages and noted that given the widespread fear of cancer, the authenticity of the patients’ perspectives could provide important reassurance for people facing similar decisions. In contrast, there was some concern that including clips (for example) of patients talking about their specific problems with insurance and other barriers to testing could be problematic unless explicit references to possible solutions were also included.

**Table:** 

**Recommended changes to be made to the video**	
“In this day and age, people are not necessary into… a 15 minute video. If you don’t make it too long, it would be helpful.” (Patient 7)	
“The folks represented should be younger just so that there can be like a connection…. When you’re talking about knowing your history… when you’re younger, you are not thinking about that.” (Patient 1)	
“One piece that would maybe be missing … is if someone has cancer, a lot of people do it [test] to help them make their treatment decisions.” (Genetic Counselor, Provider 1)	

Patients were mindful of the length of video with which people would be willing to engage. Patients did not generally feel the need for an intervention video to cover practical issues related to testing (including where to go to get tested and how testing occurs); providers were more likely to see this information as having some value. Providers also highlighted components of some of the segments where information provided was either out of date or possibly incorrect/misremembered; they advised removing patient reporting of such information from future intervention materials.

## Discussion

Findings from this study suggest a potential benefit of centering authentic patient experiences in a video intervention to increase the uptake of genetic testing among Black Americans. We interpret this as an indication of the possible acceptability and clinical utility of this approach and have moved forward with the development of a patient-centered video intervention to be included in a randomized trial. We learned that a video intervention should acknowledge the low level of trust within the Black community for science and medicine, as well as the tendency for families not to communicate about health issues—including cancer. Participants also expressed that it is important that an intervention addresses privacy concerns related to genetic data and the potential for test results to impact future health coverage.

We learned that a patient-centered video intervention is strengthened by the fact that it is real people being shown, not perfected representations of patients. Relatability and representation can also go beyond sociodemographic factors and diagnosis. Based on what we heard in this developmental phase, we decided that the video participants in the intervention would be limited to Black patients, and emphasis would be given to diverse representation by age, gender, and education. The sample would also be constructed to represent varied individual risk/cancer experiences (factors that make an individual eligible for testing).

Both providers and patients identified a need for factual information about inherited cancer risk and the processes and implications of genetic testing alongside experiential content. It was acknowledged that patients may not be best placed to deliver such information as it is usually outside of their expertise. On the other hand, having clinicians in the video might introduce unfamiliar/marginalizing medical jargon, and they may not be universally seen as trustworthy. The patients whom we spoke with had all been able to navigate testing and may therefore not have needed additional practical information. Still, patients emphasized the value of experiential connection in the video rather than simply the transmission of factual information. Thus, in this study, we heard that there may be a need for the intervention video to include resource information in addition to patient narratives.

Several patients interviewed for this study agreed to be included in the planned intervention video. The intervention video since developed includes extracts of responses to targeted questions on issues identified as valuable in the current study; people’s rationales for testing, the methods that they used to test, potential implications of testing, barriers/challenges to testing and communication about testing, and benefits of testing in relation to cancer risks within their family and community. We incorporated the suggestion to include more information about the individuals in the video to enhance viewers’ identification with the participants (including first name, age, diagnosis, testing received). Similarly, as patients discuss points of concern such as test costs, the intervention includes information provided.

In addition to the patient interviews, we provide some important medical facts and testing logistics in the intervention video. Accuracy of information (rather than how people came to a decision or how they felt about what occurred) was seen as critical, pointing to the value of having clinical perspectives as well as patient and research representation on the team building the intervention. For example, we learned that it is important to include patients talking about the concerns that they have about the cost of testing coupled with resources about how these costs can be covered.

## Conclusions

Since completion of this analysis, on the basis of our findings, we secured a small grant to support the creation of a professionally produced 10-min montage video with Black American patients who have undergone genetic testing within the past 2 years. Video structure, aesthetics, and sound quality were also mentioned in several interviews as important components to an effective intervention. Partnering the research team with a video production company to produce the video is intended to create a professional-looking product while also retaining the authenticity of the research interviews that were identified in this study as critical.

The video montage developed using insights from current study is unique as it focuses on the experiences of Black American patients who have undergone cancer genetic testing specifically as a catalyst to increase awareness and encourage other Black Americans to consider cancer genetic testing when recommended. As our next step in assessing the utility of this patient-centered video approach to communicating about genetic testing, we intend to conduct a randomized trial with Black American patients to evaluate whether it increases uptake of genetic testing.

## Data Availability

The datasets during and/or analyzed during the current study are available from the corresponding author upon reasonable request.
